# Modulation of odour-guided behaviour in mosquitoes

**DOI:** 10.1007/s00441-020-03368-6

**Published:** 2021-01-23

**Authors:** Sharon R. Hill, Rickard Ignell

**Affiliations:** Disease Vector Group, Department of Plant Protection Biology, Swedish University of Agricultural Sciences, Växtskyddsvägen 3, 23053 Alnarp, Sweden

**Keywords:** Olfaction, Neuroethology, Plant seeking, Host seeking, Oviposition

## Abstract

Mosquitoes are emerging as model systems with which to study innate behaviours through neuroethology and functional genomics. Decades of work on these disease vectors have provided a solid behavioural framework describing the distinct repertoire of predominantly odour-mediated behaviours of female mosquitoes, and their dependence on life stage (intrinsic factors) and environmental cues (extrinsic factors). The purpose of this review is to provide an overview of how intrinsic factors, including adult maturation, age, nutritional status, and infection, affect the attraction to plants and feeding on plant fluids, host seeking, blood feeding, supplemental feeding behaviours, pre-oviposition behaviour, and oviposition in female mosquitoes. With the technological advancements in the recent two decades, we have gained a better understanding of which volatile organic compounds are used by mosquitoes to recognise and discriminate among various fitness-enhancing resources, and characterised their neural and molecular correlates. In this review, we present the state of the art of the peripheral olfactory system as described by the neural physiology, functional genomics, and genetics underlying the demonstrated changes in the behavioural repertoire in female mosquitoes. The review is meant as a summary introduction to the current conceptual thinking in the field.

## Introduction

Olfaction is pertinent to the survival and reproduction of mosquitoes, as it allows for the location and assessment of resources, including food and oviposition sites. Mosquitoes have developed a complex sensory system to detect and respond to ecologically relevant volatile organic compounds (VOCs), and the occasional volatile inorganic compounds (e.g., carbon dioxide, ammonia), in their environment. Odorants are detected by olfactory sensory neurons (OSNs) found in hair-like sensilla on the antennae, maxillary palps, and proboscis (e.g., Bohbot et al. 2013; Davis 1984a; Ghaninia et al. 2019; Kwon et al. 2006; Majeed et al. 2016; Omondi et al. 2019). The VOCs penetrate the sensilla through cuticular pores or slits, and are then transported through sensillar lymph to the OSN membranes, either by diffusion or with the aid of odorant binding proteins (OBPs), where the VOCs interact with odorant receptors (Ors), ionotropic receptors (Irs), or gustatory receptors (Grs) (e.g., Carey et al. 2010; Matthews et al. 2016; Omondi et al. 2015; Omondi et al. 2019; Rinker et al. 2013). The OSN signal is then transmitted to higher olfactory centres, where the processing of olfactory along with other sensory information occurs (e.g., Ghaninia et al. 2007; Haverkamp et al. 2018; Lahondère et al. 2020). At each stage in this process, the olfactory signal is available for modulation by intrinsic factors and reflected in a change in innate behaviour, as described in other insects (Gadenne et al. 2016). Innate behaviours are genetically determined and critical to the survival of the animal. While hardwired, such behaviours are available for modulation by both internal and external factors (Tinbergen 1952).

Mosquitoes display clearly staged and separated life stages, as well as well-defined host-pathogen interaction phenotypes, for which the other main model insect, *Drosophila*, is not suitable. As such, mosquitoes provide an excellent model system in which to study the modulation of innate behaviours, as females display a distinct repertoire of behaviours depending on life stage (intrinsic factors) and environmental cues (extrinsic factors). Building on a strong behavioural framework, we are now at a stage in which we, with the assistance of advanced neurophysiological and genetic techniques, are able to establish key mosquito species as model systems (Matthews and Vosshall 2020) to investigate the roots of behavioural plasticity in depth. In this review, we provide a general perspective of how adult maturation, age, and nutritional and infection status affect the attraction to plants and feeding on plant fluids, host seeking, blood feeding, supplemental feeding behaviours, pre-oviposition behaviour, and oviposition in female mosquitoes. Furthermore, we provide a current overview of these molecular and neural correlates in the context of the intrinsically regulated behaviours of mosquitoes. The authors acknowledge that this may not be a comprehensive perspective, as there are species that deviate from the perceived norm.

## The behavioural framework

**Fig. 1:**
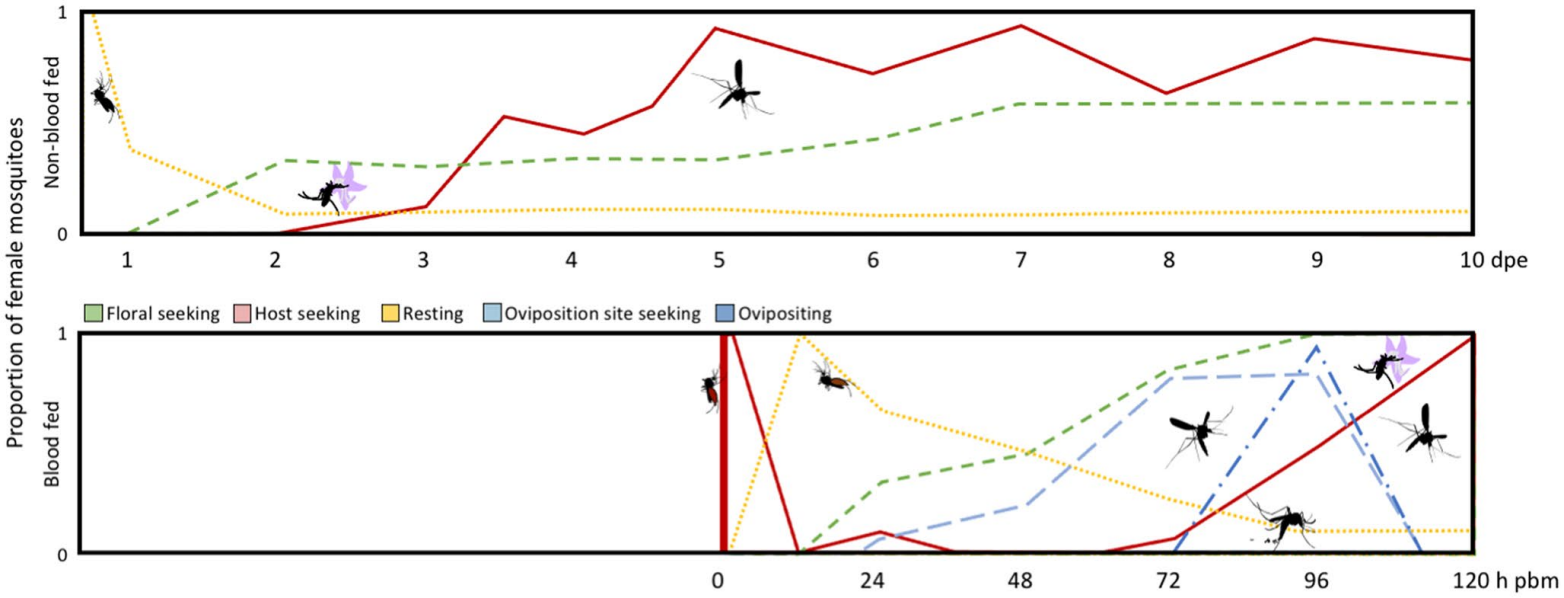
Diagrammatic representation of the adult female gonotrophic cycle. After eclosion, female mosquitoes share their activities amongst floral (green, medium-dashed line) and host seeking (red, solid line), and resting (yellow, short-dashed line), as shown over the first 10-day post-eclosion (dpe; top panel). After a full blood meal (solid red vertical bar), the floral and host seeking is inhibited, the latter until after oviposition (dark blue, dash-dot line), while the former is inhibited for up to 48-h post-blood meal (h pbm), when pre-oviposition behaviours (light blue, long-dashed line) begin (bottom panel). Most females oviposit within 96 h of a blood meal. The *y*-axis denotes the generalised proportion of mosquitoes participating in the associated behaviour based on, e.g., Alto et al. 2003; Bohbot et al. 2013; Christ et al. 2017; Davis 1984b; Foster 1995, references therein; Klowden 1989; Klowden 1994; Klowden and Blackmer 1987; Klowden and Lea 1984; and Vargo and Foster 1982

Mosquitoes depend on a series of innate behaviours to enhance survival and reproduction. While predominantly determined genetically, there is substantial experimental evidence demonstrating that these behaviours are affected by both intrinsic and extrinsic factors (Fig. 1). For reviews see Lyimo and Ferguson (2009) and Takken and Verhulst (2013). The authors strongly believe that an understanding of the behavioural framework, a set of clearly defined core innate behaviours that are based on laboratory and field observation data collected over the last decades on a plethora of species, is required to fully comprehend the factors contributing to the observed changes in odour-guided behaviours in mosquitoes. Further description of the modulation of these odour-guided behaviours in mosquitoes is found below.

### Plant attraction and feeding

The majority of mosquito species feed from floral nectaries and other sources of plant fluids, including, e.g., extrafloral nectaries, fruits, and honeydew (Barredo and DeGennaro 2020; Foster et al. 1995; Peach and Gries 2020). The prevalence of feeding on plant fluids, however, may be affected by external factors, including, e.g., differences in habitat and the seasonal availability of plant resources, which significantly increase the observed variability in plant fluid procurement by mosquitoes (Müller and Schlein 2005; Spencer et al. 2005). For a comprehensive review of this topic, see Foster (1995). The internal state also regulates the need of female mosquitoes to feed on sugar-rich plant fluids (Fig. 1). Under natural conditions, female mosquitoes often eclose with low teneral carbohydrate and lipid energy reserves, which may be rapidly replenished through feeding on plant fluids (Briegel 1990, 2003; Foster 1995; van Handel 1965). The sugars and amino acids obtained provide energy for flight (Briegel et al. 2001; Hancock and Foster 1993; Scaraffia and Wells 2003), while the carbohydrate and lipid reserves are used to enhance survival and reproduction (Briegel 1990; Briegel 2003; Manda et al. 2007; Moller-Jacobs et al. 2014; Nyasembe et al. 2020; Shapiro et al. 2016; Vrzal et al. 2010; Yu et al. 2016). As for other behaviours, feeding on plant fluids is endogenously regulated with many species displaying a diphasic feeding cycle (Chadee et al. 2014; Gillett et al. 1962, and references therein). While feeding on plant fluids declines with age, there is ample evidence that females of various species frequently feed on such resources not only during the first gonotrophic cycle but also throughout adult life (Foster 1995, and references therein; Gary and Foster 2006). However, both during adult maturation and within each gonotrophic cycle, female mosquitoes undergo sequential changes in physiological condition, which affect their avidity to sugar feed (Christ et al. 2017; Foster 1995; Gary and Foster 2006). These state-dependent changes in plant feeding are reflected in the propensity of female mosquitoes to respond to attractive plant odours that are used for both location and discrimination among plants (Foster 1995, 2008; Foster and Takken, 2004; Mauer and Rowley 1999; Nyasembe et al. 2018). In contrast, the behavioural response to repellent volatile secondary plant metabolites does not appear to be state-dependently modulated (e.g., Zappia et al. 2015), although sensitivity to repellents may reduce with age of the female (Leal et al. 2017). This suggests that there is less modulation of the detection of aversive VOCs, which likely signal risk.

Following adult emergence, female mosquitoes avidly respond behaviourally to plant-derived odours and prefer these odour sources over that of vertebrate host odours (Foster 1995; Foster and Takken 2004). As females age, and their physiological state shifts, the propensity of female mosquitoes to host seek increases (Davis 1984a; Foster and Takken 2004; Omondi et al. 2019; Tallon et al. 2019), partly driven by the energy-rich meal itself, as demonstrated in various species (Briegel 2003; Foster 1995; Hancock and Foster 1997; 2000; Renshaw et al. 1995; Roitberg et al. 2010). While domestic species, including *Anopheles gambiae* and *Aedes aegypti*, are capable of deriving all required energy from human blood, and thereby of dispensing with plant feeding, individuals from these and other species often continue to be attracted and visit plants for feeding even after developing the competence to blood feed (Barredo and DeGennaro 2020; Fernandes and Briegel 2004; Foster 1995, 2008; Hancock and Foster 1993). Flower visiting, and feeding on plant fluid, however, transiently cease after females obtain a complete blood meal, reflecting the mutual inhibition of feeding responses during consumption and digestion (Christ et al. 2017; Foster 1995). Resumption of flower visiting and sugar feeding is gradual (Christ et al. 2017; Foster, 1995), and correlates with the maturation of oocytes (Vargo and Foster, 1984), in which gravid mosquitoes and females that have recently oviposited actively seek to replenish their energy reserves by increasing their flower visiting rate (Gary and Foster 2006; Magnarelli 1977; Reisen et al. 1986).

### Host seeking and blood feeding

Anautogenous female mosquitoes, i.e., most mosquito species, require a vertebrate blood meal to complete egg maturation (Clements 1999). Recently emerged adult female mosquitoes are, however, incapable of taking a blood meal due to restricted function of their mouthparts and other organs (Clements 1999). Most anautogenous species develop the functional competence to blood feed between 24 and 72 h following adult emergence (Alto et al. 2003; Armstrong and West 1965; Clements 1999) (Fig. 1). Thereafter, blood feeding events follow an age-dependent, circadian, and diel rhythmic pattern, even in the absence of other nutrients (Alto et al. 2003; Davis 1984a; Fritz et al. 2014; Rund et al. 2016) (Fig. 1). To complete the gonotrophic cycle, female mosquitoes require a single, complete blood meal, and following this, females are generally inactive throughout the period of ovarian development and will not take another meal until after oviposition (Edman et al. 1975; Klowden and Briegel 1994; Klowden and Lea 1978; 1979a; Takken et al. 2001). This demonstrated dynamic drive to blood feed is tightly linked to the females’ propensity to host seek, i.e., respond to host odour.

While the onset of the behavioural response to host odour, and other host associated stimuli, generally co-occurs with their competence to blood feed, female mosquitoes do not display full responsiveness to host odour until between 4 to 10 days after adult emergence (Bohbot et al. 2013; Davis 1984a; Foster and Takken 2004; Omondi et al. 2015, 2019; Tallon et al. 2019), after which the proportion of host-seeking mosquitoes fluctuates around a mean maximum (ca. 90%; Alto et al. 2003; Davis 1984a). Females that have taken a full blood meal are inhibited to respond to host odour, through a two phase, immediate, and delayed process (Brown et al. 1994; Duvall et al. 2019; Klowden and Lea 1979a; 1979b). These females remain refractory to host odour, and other host associated stimuli, until after egg laying (Edman et al. 1975; Klowden and Briegel 1994; Klowden and Lea 1978; 1979a; Takken et al. 2001), at which time females actively seek new blood hosts, often after obtaining an energy-rich meal from plant fluids (Foster 1995). Such an energy-rich meal, however, appears to have no effect on the reproductive traits in anophelines, whereas it significantly reduces the overall reproductive output in *Aedes* species (Braks et al. 2006; Harrington et al. 2001).

Pathogens obtained through an infected blood meal may affect the propensity of female mosquitoes to host seek and blood feed in subsequent gonotrophic cycles, in a pathogen-specific pattern (Anderson et al. 1999; Grimstad et al. 1980; Koella et al. 1998, 2002; Platt et al. 1997; Tallon et al. 2020; Vogels et al. 2017; Wekesa et al. 1992; Yan et al. 2018). In addition, a study by Nyasembe et al. (2014) demonstrated that female *An. gambiae* infected with the malaria parasite *Plasmodium falciparum* displayed an increased attraction to floral nectar sources and sugar uptake. Moreover, Gaburro et al. (2018a, b) showed that the oviposition preference of *Ae. aegypti*, based on larval experience, was lost following dengue virus infection. These pathogen-induced changes in vector behaviour appear to be dependent on the progression of infection within the mosquito (Anderson et al. 1999; Koella et al. 2002; Tallon et al. 2020), and are often regarded as an active manipulation by the pathogen to increase transmission. However, other studies imply that these changes merely occur as a result of a general immunological response (Cator et al. 2013). The regulation of the olfactory behaviour of the mosquito vector by a pathogen requires further analysis and should be included in our discussions of vectorial capacity, as it increases the risk of human/vector interactions.

### Supplemental feeding behaviours

In addition to the gonotrophic cycling feeding behaviours described above, i.e., sugar and blood feeding, other conditions may arise that trigger intrinsic modulation of mosquito feeding to include supplementary meals. Emergence as an undernourished adult, restricted access to plant fluids and partial blood meals are among the factors that may force females to engage in bouts of multiple blood feeding to increase their fitness by both increasing energetic reserves and enhancing reproductive traits, e.g., increasing the number and/or size of offspring (Briegel and Hörler 1993; Farjana and Tuno 2013; Klowden and Briegel 1994; Scott and Takken 2012; Takken et al. 2002,  1998). This introduces variance in the observed innate behaviour, as shown by, e.g., Klowden and Briegel (1994), Klowden and Lea (1978, 1979b) and Takken et al. (2001). Interestingly, a meal of recently deposited cattle urine may substitute, at least in part, the role of a supplemental blood meal in host-seeking and gravid females (Dawit et al. 2018). Moreover, there are reports of mosquitoes being attracted to insect larvae and feeding on their haemolymph, which can result in the production of viable eggs (Harris et al. 1969; George et al. 2014; Martel et al. 2011). Whether there are other nitrogen-rich meals that play a similar role remains to be addressed. Following an incomplete blood meal, the host-seeking refractory period is shortened and host seeking resumed prior to oviposition, as oocyte development remains suspended until sufficient reserves are available (Farjana and Tuno 2013; Klowden and Briegel 1994; Klowden and Lea 1978; Takken et al. 2001). The resulting alterations in behaviour, following an incomplete blood meal, increases the interaction between the vector and human, thus have dire implications for disease transmission.

### Pre-oviposition behaviour and oviposition

The selection of an oviposition site is critical for the reproductive success of an individual female mosquito, as well as for the population dynamics of the species. Behaviours leading up to the act of oviposition, i.e., the physical deposition of eggs, are referred to as pre-oviposition behaviours (Klowden and Blackmer 1987). When mosquitoes search for a suitable egg-laying site, females are required to make choices at increasingly finer scales during orientation, encounter, and acceptance, in which water vapour and odours play significant roles (Afify and Galizia 2015; Asmare et al. 2017; Lindh et al. 2015; Okal et al. 2013; Wondwosen et al. 2016, 2017, 2018). While pre-oviposition behaviour and its regulation have not received as much attention as plant and host seeking, the response to odours associated with these behaviours, specifically those odours emanating from oviposition sites, appears to be dependent on the physiological state of the female mosquito (Klowden 1989; Klowden and Blackmer 1987; Klowden and Dutro 1990; Manda et al. 2007).

In studies on *Ae. aegypti*, in which the temporal development of the pre-oviposition behaviour has been well characterised, the onset of response to oviposition-site attractants occurs 48 h following a complete blood meal and then reaches a maximum at 72-h post-blood feeding (Klowden and Blackmer 1987) (Fig. 1). While similar studies, to our knowledge, are lacking for other mosquito species, blood meal-induced changes in the sensitivity of OSNs (Davis and Takahashi 1980; Qiu et al. 2006; Siju et al. 2010) and in the transcript abundance of genes encoding for olfactory proteins (Rinker et al. 2013) tuned to oviposition site cues suggest a similar temporal change. With the increasing identification of ecologically relevant attractants for gravid mosquitoes (Afify and Galizia 2015; Lindh et al. 2015; Okal et al. 2013; Wondwosen et al. 2016,  2017,  2018), we are now, however, in a better position to address the time line for the behavioural changes in mosquitoes, and the factors underlying modulation of the pre-oviposition behaviour.

## Sensory odour space of mosquitoes

There has been a plethora of studies on the role of odours in regulating plant-, host-, and oviposition-site seeking by female mosquitoes. For reviews see, e.g., Afify and Galizia (2015), Barredo and DeGennaro (2020), Ignell and Hill (2020), and Takken and Knols (1999). Due to recent technological advancements, and a shift in conceptual thinking concerning how insects recognise and discriminate resources, we have taken substantial strides forward in our understanding of the mechanisms regulating these behavioural traits from an ecological and evolutionary perspective (Ignell and Hill 2020). Despite the diversity of approaches taken to identify the behaviourally active VOCs in often complex odour sources, a number of key principles have emerged.

Mosquitoes exposed to synthetic VOCs presented in ecologically relevant blends or combinations generally display a stronger behavioural response than that to single VOCs (e.g., Ghaninia et al. 2019; Lahondére et al. 2020; Majeed et al. 2016; Nyasembe et al. 2018; Omondi et al. 2019; Takken and Knols 1999; Wondwosen et al. 2016). Moreover, recognition of resources, including plants, hosts, and oviposition sites, is contextual and dependent on quantitative and qualitative differences in blends and the olfactory codes evoked (e.g., Ghaninia et al. 2019; Lahondére et al. 2020; Majeed et al. 2016; Nyasembe et al. 2018; Omondi et al. 2019; Takken and Knols 1999; Wondwosen et al. 2016). In studies in which the release rate of individual VOCs within a blend has been controlled, mosquitoes have been shown to be attracted to a range of ecologically relevant doses (e.g., Ghaninia et al. 2019; Majeed et al. 2016; Omondi et al. 2019; Takken and Knols 1999; Wondwosen et al. 2016). However, at release rates higher than those encountered in the natural environment, mosquitoes are often either indifferent or averted by the same blends (Ghaninia et al. 2019; Majeed et al. 2016; Wondwosen et al. 2016, 2017, 2018). Changing the quality of the blend by changing the ratio of VOCs within it may also disrupt attraction (Ghaninia et al. 2019; Majeed et al. 2016), although, as with dose, there appears to be a range of tolerance. The addition of a VOC to a blend out of context often has a disruptive effect on blend attraction (Jaleta et al. 2016; Majeed et al. 2016). Seen from the perspective of odour quality perception, the behavioural response to any odour blend is a result of combinatorial coding, relying on the simultaneous detection and relative quantification of VOCs by the OSNs (Haverkamp et al. 2018; Lahondére et al. 2020; Wynne et al. 2020). Any change in the odour encountered, or in the signal relayed from the OSNs, will change the representation of a given stimulus in the higher olfactory centres and may lead to a change in the behavioural output (Haverkamp et al. 2018; Lahondère et al. 2020; Wynne et al. 2020).

With an increasing number of behaviourally active VOCs being identified, it is becoming clearer that these compounds are used parsimoniously, i.e., serve multiple functions in different behavioural contexts (Ignell and Hill 2020; Syed 2015). Chemical parsimony is reflected in the conserved function, in terms of selectivity and sensitivity to salient VOCs, of the peripheral olfactory system among widely divergent mosquito species. For reviews see, e.g., Afify and Galizia (2015), Barredo and DeGennaro (2020), Ignell and Hill (2020), and Takken and Knols (1999). One fundamental rule underlying chemical parsimony is that the number of VOCs available for coding is constrained by the limited number of biosynthetic pathways available for production (Blum 1996). Parsimoniously used VOCs, often in combination with other VOCs, are known to elicit stereotyped behaviours, in which the blend and ratio of VOCs convey the code. From an odour coding perspective, such reliance on the combinatorial coding of parsimoniously used VOCs provides insects with the ability to adapt to a changing intrinsic and extrinsic environment. The main selection pressure is thereby proposed to be on the maintenance of the combined code in the CNS, while the peripheral detection machinery can change (e.g., substituting ligand or receptor), so long as the pattern in the CNS response is maintained within its tolerance levels.

## Sensory and molecular correlates of olfactory behaviour

The modulation of olfactory behaviour in mosquitoes is underpinned by a highly plastic peripheral olfactory system (as described below), as opposed to that generally described in other insects (Gadenne et al. 2016). Over the past 30 years we have been accumulating physiological evidence emphasising that OSNs of mosquitoes alter activity in response to intrinsic factors (Bohbot et al. 2013; Davis 1984a, b; Eilerts et al. 2018; Grant and O’Connell 2007; Omondi et al. 2015; Omondi et al. 2019; Qiu et al. 2006; Rund et al. 2013b; Siju et al. 2010; Stanczyk et al. 2019; Tallon et al. 2020). With the advent of functional genomics, we are now starting to describe the molecular correlates that form the basis of the olfactory system regulation in mosquitoes. For past reviews on this topic see Criscione et al. (2015), Rinker et al. (2016) and Adelman et al. (2016). Here, we provide a current overview of what is known about the neural and molecular correlates underlying the demonstrated changes in the behavioural repertoire in female mosquitoes.

### Maturation and aging

**Fig. 2:**
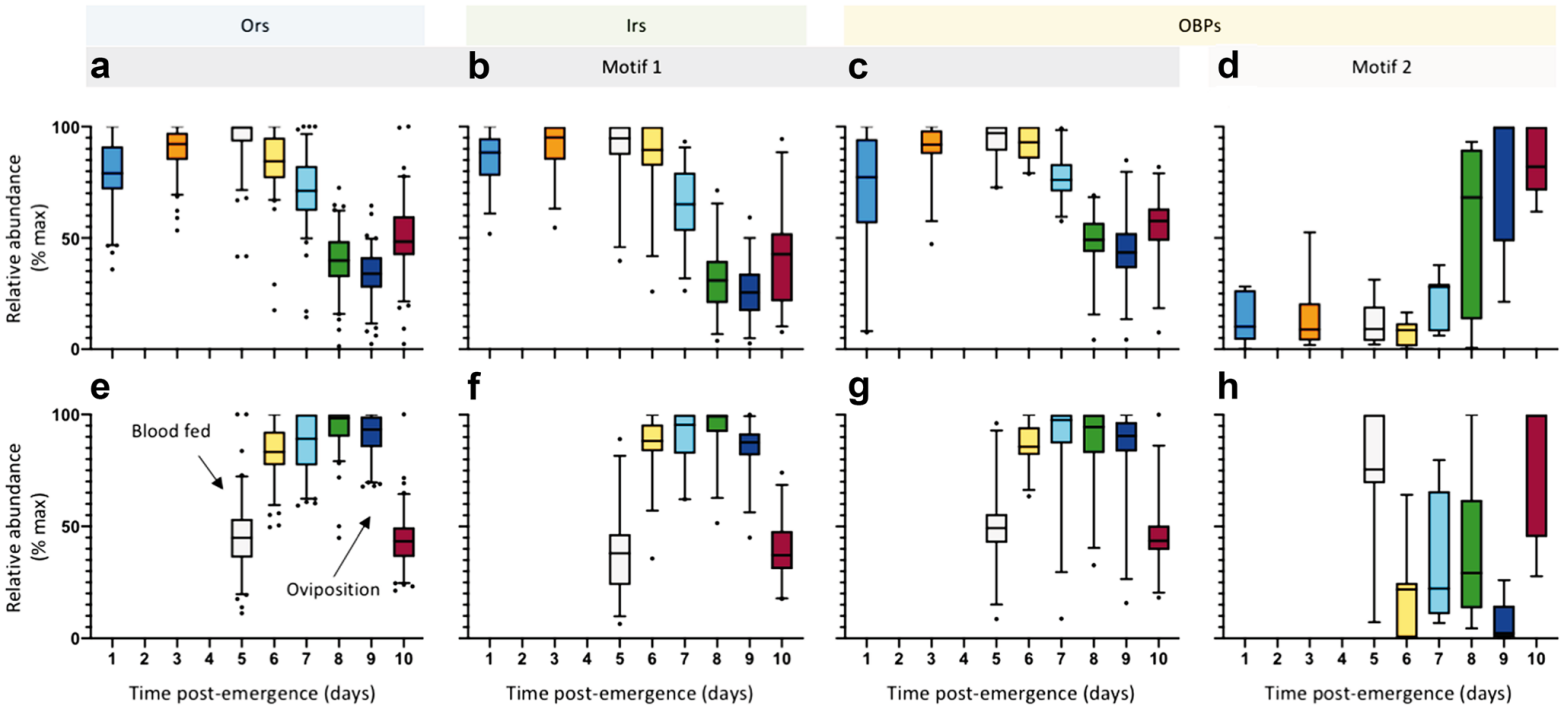
Patterns of antennal transcript abundance for select chemosensory gene families depicted during the first gonotrophic cycle of a female mosquito. The relative transcript abundance, as determined as the percent of the maximal average abundance over time (days) for each gene of the odorant receptor (Or, **a**, **e**), ionotropic receptor (Ir, **b**, **f**), and odorant binding protein (OBP, **c**, **d**, **g**, **h**) families, in the antennae of female *Aedes aegypti* are shown in box plots, with the whiskers denoting 5–95 percentile. Antennal transcript profiles were made from females with *ad libitum* access to sugar, and either no access to blood (**a**–**d**) or a complete blood meal at 5-day post-emergence (**e**–**h**). There are two evident motifs in transcript abundance, motif 1, as shown by the receptor families (**a**, **b**, **e**, **f**) and the majority of the expressed OBPs (**c**, **g**), and motif 2, as observed in the remaining 20% (**d**, **h**). Data presented here originate from Tallon et al. (2019) and Hill et al. (accepted). Note that data was not collected on days 2 and 4 for non-blood fed females, and as females were fed on day 5, no data is shown in days 1–4 for blood fed females

The sensitivity and specificity of the peripheral olfactory system in mosquitoes change through time (Bohbot et al. 2013; Davis 1984a; Grant and O’Connell 2007; Omondi et al. 2015,  2019; Qiu et al. 2006; Siju et al. 2010), reflecting, and likely underpinning, many of the odour-mediated behavioural shifts during maturation and aging. The period of maturation differs for various odour-guided behaviours, as described above. Within minutes to hours of eclosion, once the wings have unfurled and the cuticle hardened sufficiently, mosquitoes of both sexes are competent to seek for and imbibe plant fluids, but not yet for seeking a mate or, for females, a blood meal (Armstrong and West 1965; Clements 1999). As of yet, we have limited understanding of the development of the OSNs and gene expression in the peripheral olfactory system within the first 24-h post-emergence. The only study, so far, that addresses the function of OSNs within this period demonstrated that OSNs housed in grooved peg sensilla are not spontaneously active and do not respond to the host-related compound lactic acid (Davis 1984a). By 24-h post-emergence, on the other hand, these and other OSNs demonstrate spontaneous activity and sensitivity to ligands (Davis 1984a; Grant and O’Connell 2007; Omondi et al. 2015,  2019), and the majority of the genes encoding for olfactory receptors and binding proteins are expressed (Bohbot et al. 2013; Omondi et al. 2015, 2019; Tallon et al. 2019) (Fig. 2). Any gene expressed at this time point appears to be expressed throughout the adult period (Bohbot et al. 2013; Hill et al. accepted; Matthews et al. 2016; Omondi et al. 2015; Rinker et al. 2013) (Fig. 2). Only a subset of these genes is up- or down-regulated genes, and none of these appear to be switched on or off (Bohbot et al. 2013; Hill et al. accepted; Matthews et al. 2016; Omondi et al. 2015; Rinker et al. 2013). Thus, by 24 h post-emergence, the peripheral olfactory system of the adult mosquito appears to have completed the overall development of the pattern of chemosensory gene expression.

Sexual maturity follows emergence within 18–24 h for males and 1–3-day post-emergence for females (Clements 1999). While sex pheromones have not yet been identified in mosquitoes, aggregation pheromones that affect the recruitment of both sexes to the mating swarm has been shown in *Anopheles*, and candidates proposed in *Ae. aegypti* (Cabrera and Jaffe 2007; Fawaz et al. 2014; Mozũraitis et al. 2020). However, there are no molecular correlates (e.g., binding proteins or receptors) for these pheromones that have been identified. The switch from the intrinsically dominant host-seeking behaviour to mating appears to be a result of sexually mature females repressing the expression of a subset of genes involved in host seeking following their exposure to sexually mature males, which is reversed following insemination (Alonso et al. 2019; Jones 1981; Jones and Gubbins 1978).

Female mosquitoes begin to develop the ability to seek for a blood host from as young as 1 d post-emergence, and continue to mature for up to 10-day post-emergence (Alto et al. 2003; Davis 1984a; Omondi et al. 2015, 2019; Tallon et al. 2019). While both OSN sensitivity and olfactory-related gene expression generally increase as females mature to host-seeking age (Bohbot et al. 2013; Davis 1984a; Grant and O’Connell 2007; Hill et al. accepted; Omondi et al. 2015; Omondi et al. 2019; Tallon et al. 2019) (Fig. 2), there are a few notable exceptions, which has been postulated to gate the switch from pre-host seeking to host seeking (Omondi et al. 2019; Tallon et al. 2019). For example, the host responsiveness of 1-day and 4-day post-emergence female *Anopheles coluzzii* has been demonstrated to correlate with the reduction in OSN sensitivity to the host cue sulcatone and the decreased abundance of its cognate odorant receptor, *Or39*, with age (Omondi et al. 2019). Moreover, an odorant receptor, *Or117*, and two odorant binding proteins, *Obp22* and *Obp64*, with a similar expression pattern to *An. coluzzii Or39* have been described for *Ae. aegypti* (Tallon et al. 2019). While the ligand specificities of these proteins are not yet known, these may serve a similar function as proposed for *An. coluzzii*. With the availability of gene editing tools, we are now in a position to assess whether the observed expression pattern of chemosensory genes and changes in sensitivity of OSNs during maturation regulate the onset of age-dependent behaviours, e.g., host seeking and mating.

While the onset of host seeking in many anautogenous mosquito species appears to be completed within a week of eclosion (Clements 1999; Davis 1984a), the propensity to engage in the behaviour appears to oscillate as the female ages (Alto et al. 2003), as does the abundance of a subset of olfactory-related genes (Hill et al. accepted). Such oscillations may represent opportunities within the host-seeking phase of the gonotrophic cycle for the female mosquitoes to replenish dwindling energy reserves by engaging in plant-seeking activities (Alto et al. 2003; Hill et al. accepted). In general, as female mosquitoes age, the incidence of plant feeding decreases and host seeking increases (Foster 1995; Gary and Foster 2006). Few studies have investigated the sensitivity of OSNs or the transcript abundance in the peripheral olfactory system of females in older host-seeking females, and these data are largely collected as controls for assessing state-dependent change (Davis 1984a; Bohbot et al. 2013; Hill et al. accepted; Tallon et al. 2020), as there is low probability of finding older (> 10-day post-emergence) nulliparous females in the wild. Collectively, available data suggests that host seeking is regulated and modulated as the female ages, and this is, at least in part, due to changes in expression of the chemosensory-related genes in the peripheral olfactory system (Fig. 2). Future functional studies are, however, required to elucidate the molecular underpinnings of the periodic age-dependent behaviours.

### Circadian and daily rhythm effects

The cycling of odour-mediated behaviour throughout the day is reflected in the neural and molecular correlates of the peripheral nervous system in mosquitoes (Das De et al. 2018; Eilerts et al. 2018; Rund et al. 2011,  2013a, b). The sensitivity of the peripheral olfactory system to various ecologically important VOCs changes in a daily rhythm in both *An. gambiae* (Rund et al. 2013b) and *Ae. aegypti* (Eilerts et al. 2018). Of the responses to VOCs that cycle, the peripheral olfactory system is most sensitive to VOCs that induce attraction when assessed as single compounds during the peak activity periods in both species (Rund et al. 2013b; Eilerts et al. 2018). This includes the sensitivity of OSNs tuned to the ubiquitous host volatile inorganic compound, carbon dioxide (Siju, Hill and Ignell, *unpublished data*). The VOCs that are avoided when tested as single compounds, on the other hand, are detected with the highest sensitivity pre- and post-activity peak (Eilerts et al. 2018), suggesting a role for these VOCs in regulating the daily onset and cessation of odour-mediated activities. It is important to note that VOCs may display corresponding cycling of emissions, e.g., floral scent (for review see, e.g., Fenske and Imaizumi 2016). Further investigation of the cycling of OSN sensitivity to ecologically relevant VOCs, and blends thereof, at naturally fluctuating release rates, is required to fully comprehend the correlation with demonstrated rhythmic behaviours.

Cycling of chemosensory gene expression, protein translation, and degradation plays a role in generating the daily cycling in OSN sensitivity and odour-mediated behaviour of female mosquitoes (Rund et al. 2011,  2013a, b; Das De et al. 2018). The expression of at least 15 *OBP*s, 60 putative odorant degradation enzymes (*ODE*s), 4 class B scavenger receptors (*SCRB*s), including *SNMP1*, and the *Or* co-receptor (*Orco*), cycle in a daily rhythm in the heads of host-seeking *An. gambiae* females (Rund et al. 2011, 2013a). Proteomic analysis of these females’ antennae revealed 12 OBPs rhythmically cycling in protein abundance, offset from the transcript abundance cycle by 9–10 h, with 11 OBPs peaking in abundance during the scotophase period of maximal OSN sensitivity and odour-mediated activity, clearly indicating a role of OBPs in the regulation of odour-mediated daily rhythms (Rund et al. 2013b). While the transcript abundance of *Orco* cycles with a peak similar to that of the majority of the cycling *OBPs* in the heads (Rund et al. 2011, 2013a), Orco did not cycle significantly in the proteomic study of the antennae (Rund et al. 2013b). The expression level of membrane proteins in the antennae is low, and as such, Orco was the only Or to be identified (Rund et al. 2013b). Thus, further investigation is required to determine whether Ors play a role in the daily rhythm of the peripheral olfactory system and odour-driven behaviours.

Daily rhythms in olfactory-related gene expression demonstrate three types of regulation by the entrainment to light and/or by the internal molecular clock of an organism (Rund et al. 2013a). Type I genes (e.g., *OBP6*) require both the internal molecular clock and entrainment to light to maintain the daily rhythm, and when deprived of light will “run down” over time (Rund et al. 2013a). Type II genes (e.g., *OBP2*, *SNMP1*) are primarily regulated by the internal molecular clock; however, the level of expression is enhanced in the presence of light (Rund et al. 2013a). Finally, type III genes (e.g., *OBP51*) are regulated entirely by entrainment to the light, and when placed in constant darkness will immediately lose rhythmicity (Rund et al. 2013a). Several *cis*-response elements demonstrate a degree of correlation with each of the three types of rhythmic regulation underlying the function of the peripheral olfactory system of the mosquito, suggesting a complex interaction among the circadian and photic signalling pathways regulating odour-driven behaviours in mosquitoes (Rund et al. 2013a).

### The effect of a blood meal

As described above, the advent of a blood meal alters the behaviour of a female mosquito, and appears to be modulated, at least in part, by the peripheral olfactory system. A subset of antennal and maxillary palp OSNs change in sensitivity post-blood meal, both increasing and decreasing in response to a subset of cognate ligands (Davis 1984b; Hill et al. 2019; Qiu et al. 2006; Siju et al. 2010). Investigations into the molecular basis of this modulation of OSN sensitivity have assessed the abundance of antennal and maxillary palp gene transcripts, pre- and post-blood meal, in the females of several species, and have also identified subsets of blood meal regulated chemosensory genes (Hill et al. 2019; Hill et al. accepted; Matthews et al. 2016; Rinker et al. 2013; Taparia et al. 2017) (Fig. 2).

Time post-blood meal is another factor, in addition to blood meal, that modulates OSN sensitivity (Davis 1984b; Davis and Takahashi 1980; Qiu et al. 2006; Siju et al. 2010), chemosensory transcript abundance (Hill et al. accepted; Matthews et al. 2016; Rinker et al. 2013) (Fig. 2), and odour-driven behaviours. Immediately following a blood meal, few of the olfactory-related transcripts change significantly in abundance (Hill et al. accepted; Matthews et al. 2016; Rinker et al. 2013), suggesting that the cessation of host seeking after a blood meal partly relies on a limited number of olfactory-related genes in the peripheral olfactory system. Between 1- to 3-day post-blood meal, a small subset of these genes appears to be regulated (Hill et al. 2019; Hill et al. accepted; Matthews et al. 2016; Rinker et al. 2013), which may be correlated with the gradual restoration of sugar feeding (Christ et al. 2017). However, 96-h post-blood feeding, the majority of these genes in the antennae of *Ae. aegypti* follow one of two profiles, either a significantly higher (motif 1) or a significantly lower (motif 2) transcript abundance (Matthews et al. 2016; Hill et al. accepted) (Fig. 2), which correlates with the time when females are engaged in pre-oviposition behaviours. Post-oviposition, the abundance of chemosensory-related genes appears to return to host-seeking levels (Hill et al. accepted) (Fig. 2). Similar to the regulation described for the diel and circadian cycling olfactory-related genes (see above), several cis-regulatory elements correlate to each of the two age- and state-dependent motifs (Hill et al. accepted). This suggests that signalling pathways associated with the monitoring of age, feeding, and reproductive state may modulate odour-driven behaviours in mosquitoes through, as yet undescribed, transcriptional regulatory pathways.

To date, there has only been a single attempt to correlate the Or ligand sensitivity (Carey et al. 2010; Wang et al. 2010) with transcript abundance pre- and post-blood meal in *An. gambiae* females (Rinker et al. 2013), to produce a profile of the predicted change in antennal receptivity to these ligands over the first 48 h after blood feeding (Rinker et al. 2013). In looking at the antennal response as a whole, we are able to observe an immediate increase in sensitivity to one subset of VOCs post-blood meal, followed by an increase in sensitivity to another subset of compounds 24 h later (Rinker et al. 2013). The ongoing functional characterisation of Ors in various mosquito species is likely to shed light on the relevance of these changes in transcript abundance for behavioural modulation.

### Infection

Pathogen-infected mosquitoes often exhibit altered odour-mediated behaviours (Koella et al. 2002; Nyasembe et al. 2014; Gaburro et al. 2018a, b; Tallon et al. 2020), corresponding with post-infection changes in the sensitivity of the peripheral olfactory system to behaviourally relevant odorants (Stanczyk et al. 2019; Tallon et al. 2020) and neural activity patterns in the brain (Salazar et al. 2007). Moreover, the abundance of many transcripts related to neural signalling change in response to pathogen infection in the antennae, heads, and bodies of mosquitoes (Emami et al. 2017; Gaburro et al. 2018a, b; Salazar et al. 2007; Tallon et al. 2020), suggesting that neural signalling pathways may be one target of pathogen-induced modulation. In addition, a limited number of olfactory-related transcripts alter abundance in the antennae of host-seeking females capable of transmitting the pathogen (Tallon et al. 2020), indicating that the regulation of these chemosensory transcripts is another target of pathogen manipulation. Both of these changes in gene expression in response to pathogen infection may play a role in increasing the likelihood of disease transmission through the manipulation of coding in the olfactory system.

## Conclusion

From the state of the art in mosquito neuroethology and functional genomics, these insects are emerging as models for the investigation of the mechanisms underpinning behavioural plasticity. With the ongoing functional characterisation of the sensory and molecular correlates of the peripheral olfactory system, and how these affect the behaviour, in important disease vector mosquitoes, *An. gambiae *sensu lato, *Ae. aegypti*, and *Culex quinquefasciatus*, we are creating a solid foundation for further understanding of the mechanisms regulating the diverse repertoire of behaviours that dictate vectorial capacity. With such models, we are better positioned to identify novel targets for vector control.
